# Negative Impact of Sadness on Response Inhibition in Females: An Explicit Emotional Stop Signal Task fMRI Study

**DOI:** 10.3389/fnbeh.2020.00119

**Published:** 2020-07-24

**Authors:** Jianrui Ding, Yongming Wang, Chuan Wang, Federico d’Oleire Uquillas, Qinghua He, Li Cheng, Zhiling Zou

**Affiliations:** ^1^Faculty of Psychology, Southwest University, Chongqing, China; ^2^Sino-Danish College, University of Chinese Academy of Sciences, Beijing, China; ^3^Sino-Danish Center for Education and Research, Beijing, China; ^4^Department of Neurology, Massachusetts General Hospital, Harvard Medical School, Boston, MA, United States; ^5^Princeton Neuroscience Institute, Princeton University, Princeton, NJ, United States; ^6^Faculty of Education, Beijing Normal University, Beijing, China

**Keywords:** sadness, response inhibition, emotional stop signal task, functional magnetic resonance imaging, superior frontal gyrus

## Abstract

Response inhibition is a critical cognitive ability underlying executive control over reactions to external cues, or inner requirements. Previous studies suggest that high arousal negative emotions (e.g., anger or fear) could impair response inhibition in implicit emotional stop signal tasks (eSSTs). However, studies exploring how low arousal negative emotions (e.g., sadness) influence response inhibition remain sparse. In the current study, 20 female college students performed an explicit eSST to explore the influence of sadness on response inhibition and its neural mechanism. Participants are instructed to press a button to sad or neutral facial stimuli while inhibiting their response during the presentation of a stop signal. Results showed that compared with neutral stimuli, sad stimuli were related to increased stop signal reaction time (SSRT) (i.e., worse response inhibition). Compared with neutral condition, higher activation during sad condition was found within the right superior frontal gyrus (SFG), right insula, right middle cingulate cortex (MCC), bilateral superior temporal gyrus (STG), left lingual gyrus, and right motor cortex. These findings indicated that sadness, like other negative emotions, may impair response inhibition in an explicit way and highlight the explicit eSST as a new paradigm to investigate the subtle interaction between negative emotion processing and cognitive control.

## Introduction

Response inhibition is a critical cognitive ability underlying executive control over reactions to external cues, or inner requirements, so as to enhance fitness to the natural environment (Congdon et al., 2012). Many negative emotionality (Pawliczek et al., 2013), personality traits like impulsivity (Lawrence et al., 2009), and many diseases such as depressive disorder (Dai et al., 2011), schizophrenia (Gopin et al., 2011), attention deficit hyperactivity disorder (Aron and Poldrack, 2005) have a close relationship with inhibitory ability injury.

Negative emotions (e.g., fear and anger) have been found to impair response inhibition, which are always studied with the emotional Stop Signal Task (eSST) (Kalanthroff et al., 2013; Rebetez et al., 2015; Patterson et al., 2016), in which the stop signal reaction time (SSRT) is used as the main index of response inhibition ability. Negative stimuli are associated with longer SSRTs which represent a worse response inhibition, indicating an “interruption effect.” This effect has been attributed to the increased attention and deeper processing of emotional stimuli relative to non-emotional stimuli (Patterson et al., 2016).

However, the interruption effects of negative emotions are mostly based on studies of high-arousal negative emotions, such as fear and anger. None of those studies examined the specific influence of low-arousal negative emotion (e.g., sadness), which is one of the main purposes of the current study.

Sadness, the individual experience in an unpleasant situation and inner feeling of losing or no gain, is a negative-valence, low-arousal basic emotion and more far-ranging, longer lasting, more meaningful in daily life relative to other emotion states (Ellsworth and Smith, 1988). Its motivation should be self-protection and avoid unpleasant context (Albert et al., 2010). When human beings deal with sadness, some cerebral limbic and paralimbic systems including ventral medial prefrontal cortex (vmPFC) (Suslow et al., 2013); anterior cingulate cortex (ACC) (Habel et al., 2005); anterior temporal cortex, insula (Liotti et al., 2000); amygdala (Aldhafeeri et al., 2012); precuneus, hypothalamus; and pons, midbrain come into activation (Lévesque et al., 2003).

Besides the fact that sadness has seldom been examined in this field, previous eSST studies have used different variations of the eSST to explore the interaction of emotion and response inhibition. In some studies, emotional stimuli precede the inhibition of neutral stop targets (Verbruggen and De Houwer, 2007; Kalanthroff et al., 2013; Yu et al., 2015; Patterson et al., 2016) (referred to as a “priming eSST,” as the emotional stimuli are shown prior to neutral stop targets). In other studies, however, negative facial expressions are used as targets for response inhibition (Sagaspe et al., 2011; Pawliczek et al., 2013; Rebetez et al., 2015; Derntl and Habel, 2016), in which participants are either asked to categorize the gender of emotional face stimuli (Sagaspe et al., 2011; Rebetez et al., 2015) or to perform a simple “go” reaction time task regardless of the type of emotion displayed (Pawliczek et al., 2013; Derntl and Habel, 2016) while withholding their response whenever stop signals appear. Because the processing of the emotional stimuli of interest occurs outside of the scope of deliberate attention, and are incidentally processed, we refer to it as “implicit eSST.” As such, both “priming eSST” and “implicit eSST” may fail to test motoric inhibition during intentional and conscious executive processing of emotional stimuli. However, in daily socio-emotional contexts, in order to form and maintain an intimate relationship especially in the early stage of love, it is of great importance executing successful response inhibition over negative emotions in an explicit manner (Song et al., 2016). For example, a boy would stop complaining once he noticed sadness on his girlfriend’s facial expression. Thus, in our recent study, we for the first time used facial expressions as the targets of response inhibition (Song et al., 2016), in which participants were required to respond according to the type of emotion shown on the face stimuli (sad vs. neutral) and to stop any reaction when a stop signal (a red cross mark) occurred. In the interest of parsimony, we referred to this paradigm as an “explicit eSST,” as participants had to intentionally process facial emotions while simultaneously being engaged in the stop signal task (SST). We found a significant main effect of emotion with longer SSRT in the sad condition. These findings showed that sadness, like other negative emotions, may also interfere with response inhibition and at the same time suggested that explicit eSST could be a good paradigm to explore subtle differences in the field of the interaction between emotion and cognitive control. Thus, in the present study, we aimed to confirm again the negative influence of sad facial expressions on response inhibition using an explicit eSST and, furthermore, to explore the neural correlates of this process with functional magnetic resonance imaging (fMRI).

A recent fMRI meta-analysis indicated that response inhibition is not a unidimensional construct but consists of subcategories of cognitive processes that engage common as well as distinct neural correlates and networks (Zhang et al., 2017). A study exploring the neural mechanisms of generic response inhibition has revealed the important role of the fronto-basal ganglia-dependent system, including the right inferior frontal cortex (IFC), subthalamic nucleus (STN), pre-supplementary motor area (pre-SMA), striatum (STR), substantia nigra, and globus pallidus (for review, see Nambu et al., 2002; Rae et al., 2014; Zhang et al., 2017). However, previous studies on the neural mechanisms of the interaction between negative emotion and response inhibition remain sparse. There were two studies combining fMRI with an eSST. A study by Sagaspe et al. (2011) revealed no changes in SSRT with fearful expressions, while on a neural level, their results suggested that the amygdala played a role in the influence of fear on motoric inhibition *via* direct or indirect functional interplay with motor pathways. Pawliczek et al. (2013) found a response facilitation effect with angry faces during stop trials and higher activity in the limbic system (i.e., the amygdala, motor, and somatosensory areas). Altogether, the fronto-basal ganglia-dependent system and the limbic system seemed to be the most important areas underlying the effects of negative emotions.

Based on those previous findings, we hypothesized that the emotional stimuli would pose as a distractor compared with neutral stimuli, and there would be a prolonged response inhibition (longer SSRT) for sad trials compared to neutral trials. We also hypothesized that response inhibition in sad conditions would be associated with a higher activation in the frontal cortex and limbic system.

## Materials and Methods

### Participants

Twenty-two healthy female college students (mean age: 21.20 ± 1.70 years) were randomly recruited from the Southwest University (SWU, Chongqing, China) campus by flyer or Internet-based advertisement. They all were right-handed, native Chinese speakers and had normal or corrected to normal vision. All participants were informed that their participation was completely voluntary and that they may withdraw from the study at any time. A written informed consent was obtained from each participant, and this research was approved by the Ethics Committee of SWU.

Only female participants were enrolled, as studies demonstrate heightened neural activity to emotional stimuli in women relative to men (Goldin et al., 2008; Crowell et al., 2014). In addition, studies have demonstrated that females and males may have somewhat distinct neural mechanisms underlying the processing of emotional faces (Lee et al., 2002), as well as response inhibition (Li et al., 2006, 2009; Thakkar et al., 2014). Thus, we explored the neural mechanism of the interaction of sadness and inhibition with female for the first step, and gender difference could be the next future research question.

### Functional Task

**FIGURE 1:**
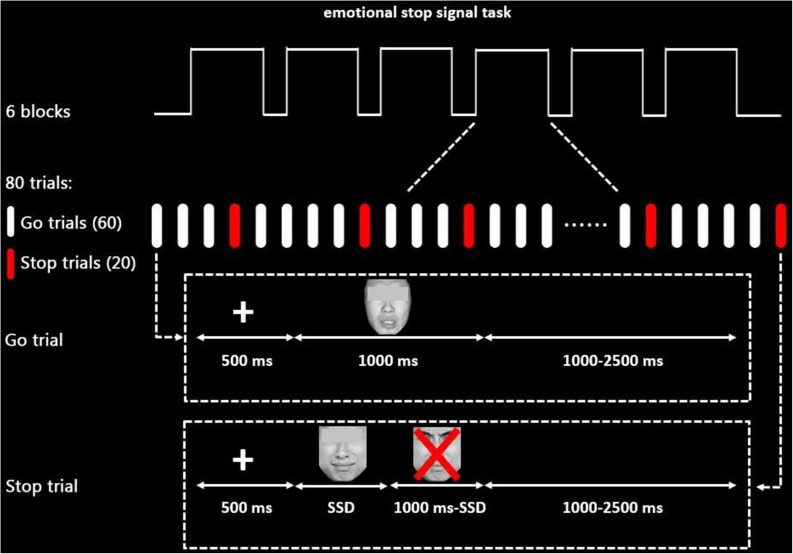
Schematic display of the emotional stop signal task paradigm (sad stimuli as an example). The eyes of face pictures that participants see are not obscured in each trial.

An explicit eSST (Figure 1) based on previous works (Song et al., 2016) was used in the current study. The stimuli comprised of 80 facial expression pictures, half of which were sad and half were neutral. All pictures were taken from the Chinese Affective Face Picture System (Luo et al., 2010) (male and female face pictures in half). The valence, arousal, and attractiveness of each picture were rated by 40 SWU students who are independent from the sample participating in the main study (mean age: 22.1 ± 1.90 years). For valence ratings (mean: sad = 2.85 ± 0.56, neutral = 4.30 ± 0.44), sad pictures were rated as more negative [*t*(78) = −12.97, *p* < 0.001]; for arousal ratings (mean: sad = 5.30 ± 0.64, neutral = 4.04 ± 1.15), sad pictures were rated as more arousing [*t*(78) = 6.04, *p* < 0.001]; for attractiveness ratings, there were no significant differences between sad and neutral pictures [*t*(78) = −1.29, *p* > 0.05]. All pictures were similar in their size, brightness, contrast grade, background, visual angle, and resolution ratio.

The task paradigm was programmed using the E-Prime package (Psychological Software Tools, Inc., Pittsburgh, PA, United States), consisting of 480 trials divided into six blocks equally, 360 of which were go trials, while 120 were stop trials in a pseudorandom order with a maximum of two stop trials in sequence. There were 1-min breaks between blocks to allow participants to relax.

In each trial, a 500-ms fixation was followed by an emotional stimulus (1,000 ms). Participants were instructed to differentiate the emotion of the facial expression and responded by pressing “1” for sad emotion and “2” for neutral emotion (keyboard buttons were counterbalanced among subjects) as quickly and accurately as possible during the 1,000 ms (go trials). Importantly, they were also required to inhibit their response if a red “X” mark occurred after face stimuli (stop trials). A trial was either terminated by pressing the button or when times exceeded 1,000 ms.

To obtain about 50% inhibition accuracy rate for both neutral and sad trials, the interval between the stimuli onset and the occurrence of the stop signal [stop signal delay (SSD)] was adjusted automatically based on a previous stop trial. The SSD for the first stop signal was set to 250 ms. Then, 50 ms was added if the participants stop successfully (or subtracted if they failed), thereby making it harder (or easier) in the following stop trials to obtain a 50% inhibition accuracy rate for stop trials. The interstimulus interval was jittered from 1,000 to 2,500 ms to ensure that the hemodynamic response function (HRF) was sampled at different volumes after stimulus onsets. Participants were required to practice several experimental trials before entering the scanner until their accuracy was higher than 85% in the go trials.

### Functional MRI Acquisition Parameters

All imaging data were acquired using a 3-tesla Siemens MAGNETOM Tim Trio whole-body magnetic resonance scanner (Siemens, Germany) at the Brain Imaging Research Center of the SWU. Individuals’ head movements were minimized by cushions. Stimuli were displayed on a screen placed at the rear of the scanner and could be observed by a mirror that was mounted on the imaging head coil.

Functional data were acquired using a T2-weighted gradient echo planar imaging (EPI) sequence: repetition time (TR) = 2,000 ms, echo time (TE) = 30 ms, field of view (FOV) = 220 mm^2^ × 220 mm^2^, slices = 32, slice thickness = 3 mm, voxel size = 3.4 mm × 3.4 mm × 3 mm, flip angle = 90°, matrix resolution = 64 × 64. After functional neuroimaging, T1-weighted anatomical images were collected: TR = 1,900 ms, TE = 2.52 ms, FOV = 256 mm^2^ × 256 mm^2^, slices = 176, slice thickness = 1 mm, voxel size = 1 mm × 1 mm × 1 mm, flip angle = 90°, matrix resolution = 256 × 256. Corresponding to the task, all scan procedures included six runs, and every run took 4.50 min.

### Behavioral Data Analysis

Statistical analyses were done in SPSS 22.0 (SPSS Inc., United States) with the significance level set at *p* < 0.05 (two-tailed). The data were analyzed using paired *t*-tests.

Correct go reaction time (GoRT) in go trials and error stop response reaction time (SRRT) in stop trials were recorded. Critically, the key index, the SSRT, was calculated by subtracting the average SSD from the median GoRT (for a detailed explanation, see Logan and Cowan, 1984; Pawliczek et al., 2013).

### Data Preprocessing and Analysis

Functional imaging data were preprocessed and analyzed by Statistical Parametric Mapping software (SPM12)^1^ in MATLAB R2014a (MathWorks Inc., United States). The following procedures were performed: removing the first five time points; slice-timing correction; functional realignment excluding subjects with head motion >2 mm or 2° (two participants were excluded, so all behavioral and fMRI data were based on 20 participants); normalization to standard stereotaxic space [Montreal Neurological Institute (MNI)] (interpolation to a resolution of 3 mm^3^ × 3 mm^3^ × 3 mm^3^); spatial smoothing with an 8-mm Full width half maximum (FWHM) Gaussian kernel.

After preprocessing, the individual first-level (fixed-effects) analysis was performed using separate general linear models (GLMs) for 20 female data. The model included regressors for “fixation-cross” baseline, succeeded go trials of sadness (SGS), succeeded go trials of neutral stimuli (SGN), succeeded stop trials of sadness (SSS), succeeded stop trials of neutral stimuli (SSN), and corresponding wrong trials, respectively. Further, the six realignment parameters were excluded as extraneous variables in the first-level analysis, and the activation differences of sad condition in brain regions were examined by T-contrast between go conditions (go in SGS – go in SGN) and stop conditions [(stop in SSS – go in SGS) – (stop in SSN – go in SGN)] in order to detect the significant effect of sadness on individuals’ execution and inhibitory functions, respectively. The data were high-pass filtering with a cutoff frequency of 1/128 Hz to remove low-frequency drifts.

In order to assess group differences, contrast images from all subjects were included in the second-level random-effects analysis. Activation differences in brain regions were examined by one-sample *t*-test with go conditions and stop conditions in order to detect significant sadness groups effects (Boehler et al., 2011; Fauth-Bühler et al., 2012). In addition, correlational analyses between brain activity and SSRT in the SSS and SSN conditions were conducted independently.

All contrasts were set at cluster-based FWE 0.05 correction (cluster size threshold: 50 voxels) implemented in SPM12. All resulting visualizations used BrainNet Viewer (Xia et al., 2013).

## Results

### Behavioral Data

Behavioral data of 20 participants collected during the six fMRI runs were included in a paired *t*-test with emotion (sad, neutral) as within-subjects factor. The results indicated that participants had significantly longer SSRTs on sad condition trials relative to neutral condition trials [*t*(19) = 3.13, *p* < 0.01]. No significant differences were found in correct GoRT and SRRT (Table 1).

**TABLE 1:** Behavioral performance on the emotional SST.

		**Mean**	**SD**	***t***	***p***	***Cohen’s d***
GoRT (ms)	Sadness	553.60	45.40			
	Neutral	562.51	42.08	–1.34	0.20	–0.20
SRRT (ms)	Sadness	523.04	43.34			
	Neutral	534.54	45.57	–1.68	0.11	–0.25
SSRT (ms)	Sadness	253.37	41.38			
	Neutral	238.74	37.90	3.13	**0.01**	0.37

*SST, stop signal task; GoRT, go reaction time; SRRT, stop response reaction time; SSRT, stop signal reaction time; SD, standard deviation; ms, milliseconds. Significant effects are marked in bold.*

### Functional Activation Data

**FIGURE 2:**
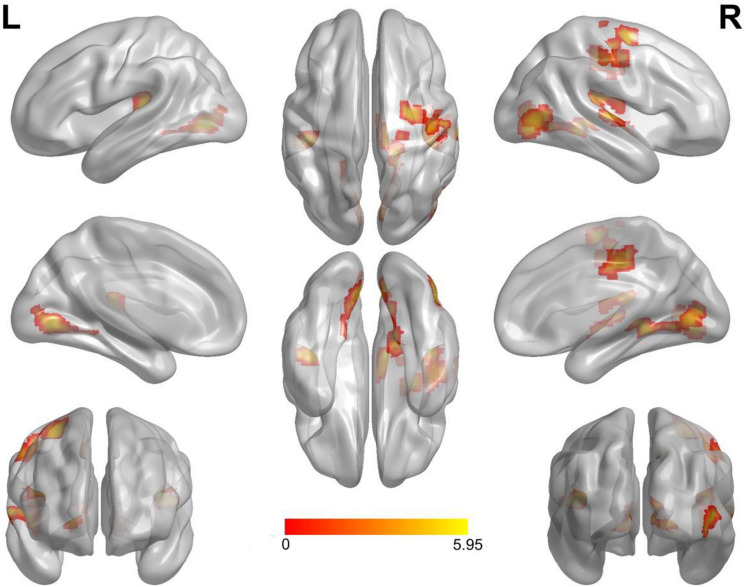
Notable brain areas showing activation differences between sad and neutral conditions in succeeded stop trials (sad > neutral). Compared with neutral conditions, sad conditions revealed remarkably increased activation in the right superior frontal gyrus, the right insula, the right middle cingulate cortex, the right precentral gyrus, the right postcentral gyrus, and the right superior temporal gyrus. There were no significantly decreased activated regions. Activation of the left hemisphere did not reach the threshold of 50 voxels (superior temporal gyrus: 44 voxels, lingual gyrus: 45 voxels). L, left. R, right.

Compared with the neutral stop condition (stop in SSN – go in SGN), increased activation was found for sad stop condition (stop in SSS – go in SGS) in the right superior frontal gyrus (SFG), right insula, right middle cingulate cortex (MCC), right precentral gyrus (PreCG), right postcentral gyrus (PostCG), and right superior temporal gyrus (STG) (Table 2 and Figure 2). There were no other significant results.

**TABLE 2:** Notable activation differences between sad and neutral conditions (sad > neutral, cluster-wise FWE 0.05 correction).

**Hemisphere**	**Region**	**Peak MNI coordinates**			**Voxels**	***t* (*p*)**
		**X**	**Y**	**Z**		
Right	SFG	21	−12	60	61	5.07 (0.017)
	Insula	39	−6	−9	91	4.54 (0.016)
	MCC	9	−15	33	88	5.38 (0.032)
	PreCG	39	−18	39	77	4.05 (0.006)
	PostCG	48	−21	39	87	3.98 (0.036)
	STG	45	−21	3	87	4.69 (0.003)
Left	STG	−48	−27	15	44	5.36 (0.052)
	LG	−9	−75	3	74	5.12 (0.001)

*MNI, Montreal Neurological Institute; SFG, superior frontal gyrus; MCC, middle cingulate cortex; PreCG, precentral gyrus; PostCG, postcentral gyrus; STG, superior temporal gyrus; LG, lingual gyrus.*

## Discussion

Here, we explored the influence of sadness on response inhibition and investigated its neural correlates using an explicit emotional stop signal fMRI task. Behavioral results confirmed that participants had significantly longer SSRTs for sad conditions relative to neutral conditions. fMRI results revealed that compared with neutral stop conditions, increased brain activation was found during sad stop conditions in the SFG, the insula, the MCC, the PreCG, the PostCG, and the STG (all right lateralized).

### The Explicit Emotional Stop Signal Task and Its Potential Application

The influence of negative emotions on cognitive control was always studied with the eSST. The eSST is a variation of the SST, a behavioral task traditionally used to measure inhibition of an action (i.e., prepotent responses) (Logan et al., 1997). However, we can find that different versions of the eSST exist, which may be a possible explanation for discordant results found in the literature regarding the interaction between negative emotion and response inhibition.

In some studies, emotional stimuli are displayed just before neutral targets, and participants are instructed to do the traditional SST while neglecting the emotional stimuli shown before (Kalanthroff et al., 2013; Patterson et al., 2016). We identify these tasks as “priming eSST” because the emotional stimuli are displayed prior to stop targets. Results from these “priming eSST” studies have demonstrated that negative stimuli are associated with worse response inhibition, which is an “interruption effect.”

Other studies used emotional stimuli as targets for response inhibition (Sagaspe et al., 2011; Pawliczek et al., 2013; Derntl and Habel, 2016). In those studies, negative facial expressions (fear, anger, etc.) were shown on the screen, and participants either categorized the sex of an emotional face stimuli (Sagaspe et al., 2011; Rebetez et al., 2015) or performed a simple “go” reaction regardless of the type of emotion displayed while inhibiting their response whenever stop signals appeared (Pawliczek et al., 2013; Derntl and Habel, 2016). We identify these kinds of paradigms as “implicit eSST” because emotional information of the stimuli (i.e., anger, sad, etc.) are incidentally processed.

There are inconsistent results found within studies using implicit eSST. Specifically, a facilitation effect from anger stimuli on response inhibition has been shown in implicit eSST involving a simple go reaction time task (Pawliczek et al., 2013; Derntl and Habel, 2016). In parallel, an interruption effect (Rebetez et al., 2015), or a lack thereof (Sagaspe et al., 2011), has been shown in studies involving gender discrimination of emotional face stimuli. These inconsistencies may be due to differences in task difficulty across studies. For example, easier go tasks (e.g., simple go reaction time task) may induce a facilitation effect, while more difficult go tasks (e.g., gender discrimination) may induce an interruption effect. Thus, it seems that eSST is a kind of susceptible task that needs to be explored in depth.

Here we used an explicit eSST. The explicit eSST looks similar but very different from the “implicit eSST” used in previous studies (Sagaspe et al., 2011; Pawliczek et al., 2013). Compared with the tasks in implicit eSST which only require a simple “go” reaction or gender discrimination of emotional face stimuli, tasks in “explicit eSST” (i.e., discriminating the emotion of face) involve intentional emotion processing and thus are more difficult and possibly induce interruption effect on cognitive processing.

The explicit eSST may have a better ecological validity than implicit eSST in some situations. In daily socio-emotional contexts, it is of great importance executing successful response inhibition over negative emotions in an explicit manner in order to form and maintain an intimate relationship especially in early stages of love (Song et al., 2016). Neither “priming eSST” nor “implicit eSST” paradigms seemed to be able to properly simulate the situations where executing inhibition with emotions is processed intentionally at the same time.

In our experiment, the interference effect that negative emotion seemed to have on inhibitory control was found when sadness was used as the negative stimuli, despite this emotion elicits low levels of arousal, which has not been reported yet with any implicit eSSTs. Our results verified aspects of the validity (probably interference effect of negative emotions) and sensitivity (even with the low-arousal negative emotion, i.e., sadness) of the explicit eSST paradigm. Thus, we believe that the explicit eSST paradigm used in the current study may be a useful paradigm in clinical settings and help in developing and tailoring of treatment interventions for those suffering from deficits of response inhibition and high impulsive traits, especially in negative emotion conditions, and may help better inform the evaluation of clinical intervention measures.

### The Influence of Sad Expressions on Response Inhibition

Sadness is a kind of negative emotion; it occurs when an individual experiences unpleasant situations or inner feelings of loss (Chepenik et al., 2007). However, it has the lowest levels of arousal among five basic negative emotions (anger, fear, surprise, disgust, and sadness). Sadness has a wider range of contexts and can be felt for longer periods of time than other emotions (Ellsworth and Smith, 1988) and can even contribute to clinical depression if left unaddressed (Goldin et al., 2008), making it crucial to understand and study.

It has long been discussed whether the role of emotion on cognitive control is due to valence (negative or positive) or arousal levels (low or high). Some research suggests that it may be emotional valence that is the main modulatory factor of cognition (Peng et al., 2014). In that framework, stimuli with negative valence should result in more alertness and focusing of attention, prolonging the stop reaction time (Rebetez et al., 2015). Nevertheless, other research argues that arousal may play the leading role, as it has been noted that the higher the arousal that stimuli have, the greater the impairment in inhibitory speed (Schimmack and Derryberry, 2005; Verbruggen and De Houwer, 2007).

In the present study, we duplicate our previous main finding (Song et al., 2016). Individuals exhibited longer SSRTs for sad condition trials as compared with neutral condition trials, suggesting impaired response inhibition in response to sad facial expression processing, which indicated that sadness (despite being an emotion of low arousal), like other strong negative emotions, can disturb behavioral inhibitory control.

We found an impairment effect associated with sadness on response inhibition, further emphasizing the importance of valence over arousal, to some extent. Future studies could, however, further explore this question by directly comparing the influence of sad stimuli with other negative emotional stimuli (e.g., fear or anger) on response inhibition within the same individual.

### The Brain Mechanisms of the Interaction Between Sadness and Response Inhibition

As far as we know, this study was the first to investigate the neural correlates underlying the interaction between sadness and response inhibition on an explicit eSST. fMRI results revealed that compared to neutral stop conditions, sad stop conditions were associated with greater activation in the SFG, the insula, the MCC, the PostCG, the PreCG, and the STG. Combined with behavioral results (longer SSRT but similar GoRT in sad conditions), our data indicated that sad stimuli specifically modulated the neural activity of inhibitory efforts, rather than “go” efforts in the eSST. These brain regions are associated with the monitoring or regulation of emotion and inhibition control.

For example, the SFG may be involved in different higher order cognitive functions, including top-down attentional control (Hopfinger et al., 2000), working memory and inhibitory control (Zhang et al., 2012), conscious decision-making and reasoning (Chase et al., 2011), and the modeling and prediction of others’ behaviors (Cui et al., 2012). In our study, higher activation of the right SFG during inhibition efforts for sad trials emphasized the importance of this region for the interplay between inhibition effort and the processing of negative (low arousal) emotional stimuli.

An fMRI meta-analysis indicated that the activation of the insula and the MCC was common across the cognitive processes (Zhang et al., 2017). The insula has been implicated in emotion processing (Singer et al., 2009), attentional control (Menon and Uddin, 2010), and interoceptive awareness (Singer et al., 2009). Previous studies have also indicated that the insula can be activated by the feeling of disgust (Wicker et al., 2003) and in happy vs. sad emotion-related tasks (Damasio et al., 2000). Furthermore, the monitoring of the internal emotional state and the successful integration of sensory and emotional information may be crucial for executing behavioral responses (Hanlon and Canterberry, 2012). At the same time, the MCC displays functional connectivity with sensory–motor areas and is often described as the cingulate motor zone (Mayka et al., 2006). A meta-analysis has shown that the MCC is active in tasks associated with memory, visual attention, and motor control (Torta and Cauda, 2011). These two regions were implicated in our study and may point to their importance in the interface between internal feelings of sadness and regulation of negative emotion.

The PreCG and PostCG may help process and integrate somatosensory and somatic motor information (Christova et al., 2013). Their increased activity in the current study during sad condition trials may represent a “readiness” toward execution of motoric action for rapid and appropriate responses. Lastly, the STG has been associated with face and voice processing (Ethofer et al., 2013). The increased STG activation in sad condition trials may suggest deeper interoceptive processing for sad faces.

In addition, compared with neutral condition trials, we did not find increased activation of the amygdala during sad inhibitory condition trials, a region well-known to function as a threat detector that has been shown to be activated in fMRI studies involving stimuli of various negative emotions (Fusar-Poli et al., 2009). Possible ways to reconcile the discrepancies between previous studies and ours could be that the activation of the amygdala during cognitive processes is regulated by the arousal level of emotional stimuli, at least in clinically normal individuals (Killgore and Yurgelun-Todd, 2004; Sagaspe et al., 2011). Or perhaps discrepancies may be better explained by implicit emotional processing rather than explicit processing (Critchley et al., 2000). Thus, more studies are needed to explore and verify the involvement of the amygdala in response inhibition during negative emotional fMRI trials of explicit eSST paradigms.

Further, there was no difference in IFG activation between the two stop conditions in our study, although the IFG has been considered as an important region for response inhibition (Rae et al., 2014; Zhang et al., 2017). It may be due to the fact that the IFG was activated for stopping processes both in the sad and neutral conditions compared with the baseline (see Supplementary Material). In addition, a previous study also suggested that the IFG was not modulated by affective factors during inhibitory control (Sagaspe et al., 2011).

### Limitations and Future Directions

There were several limitations worth mentioning. First, only females were selected in the current study, and thus conclusions may be female specific. Future studies may wish to replicate the current results in larger cohorts and take gender difference into consideration. Thus, assessing gender differences could be a next step in the development of more effective and personalized medicine preventive measures and interventions for affective and cognitive dysfunction. Second, sadness as it is measured in a laboratory setting may not fully tap into the idiosyncrasies implicated with the complex emotion of sadness as it is found in the natural environment. Thus, how to present sad stimuli that more closely resemble real-life situations is an issue that requires further development. Third, even though sadness is a low arousal emotion, the mean arousal rating of sad stimuli was still higher than neutral stimuli in the present study. Therefore, brain activation differences between sad and neutral conditions are not only attributable to sadness. And we did not compare the difference between explicit eSST and implicit eSST directly, which could be helpful for better understanding the influence of negative emotions on inhibition control. Last but not least, more data analysis methods could be tried to do to explore in depth the neural mechanism underlying the influence of sadness on response inhibition, such as generalized PPI (gPPI), graph-theoretical methods.

## Conclusion

We used an explicit eSST paradigm to explore the influence of sadness on response inhibition as well as its underlying brain correlates in female college students. We found that compared with neutral conditions, worse response inhibition (longer SSRT) was found for sad condition trials. Sad condition trials also increased brain activation in regions implicated with emotion processing, response inhibition, and interception. These findings provided evidence that sadness, like other negative emotions, had a negative effect on inhibitory processes and suggested that the explicit eSST could be a useful paradigm to investigate the subtle interaction between emotion processing and cognitive control.

## Data Availability Statement

The datasets generated for this study are available on request to the corresponding authors.

## Ethics Statement

The studies involving human participants were reviewed and approved by the Ethics Committee of Southwest University. The patients/participants provided their written informed consent to participate in this study.

## Author Contributions

ZZ and LC were responsible for the original experimental design. JD, YW, CW, Fd’O, and QH performed the data analysis, interpreted the findings, and wrote the manuscript. JD, ZZ, Fd’O, and LC revised the manuscript. All authors contributed to the article and approved the submitted version.

## Conflict of Interest

The authors declare that the research was conducted in the absence of any commercial or financial relationships that could be construed as a potential conflict of interest.

## Footnotes

1 http://www.fil.ion.ucl.ac.uk/spm
