# Supplementary material for: Negative Impact of Sadness on Response Inhibition in Females: An Explicit Emotional Stop Signal Task fMRI Study
Source: Front Behav Neurosci. 2020 Jul 24;14:119. doi: 10.3389/fnbeh.2020.00119 (PMC7396530; doi:10.3389/fnbeh.2020.00119)
Supplement: Supplementary file 1 [file Table_1.DOCX]

Table S1. Notable activation differences between successful sad stop and failed sad stop trials (*p* < 0.001 with cluster size > 50; *p*FWE values are after cluster-based FWE 0.05 correction).

| Hemisphere | Region | Peak MNI coordinates | | | Voxels | *t* | *pFWE* |
| --- | --- | --- | --- | --- | --- | --- | --- |
|  |  | X | Y | Z |  |  |  |
| R | MCC | 3 | -36 | 39 | 71 | *5.15* | *0.04* |
| R | SFG | 15 | 45 | 3 | 66 | *4.44* | *0.05* |
| L | SMG | -57 | -24 | 42 | 294 | *-5.60* | *0.001* |

MNI, Montreal Neurological Institute; MCC, middle cingulate cortex; SFG, superior frontal gyrus; SMG, supramarginal gyrus; L, left; R, right.

Figure S1. Notable brain areas showing activation differences between successful sad stop and failed sad stop trials (Cluster-based FWE 0.05 correction with cluster size > 50).


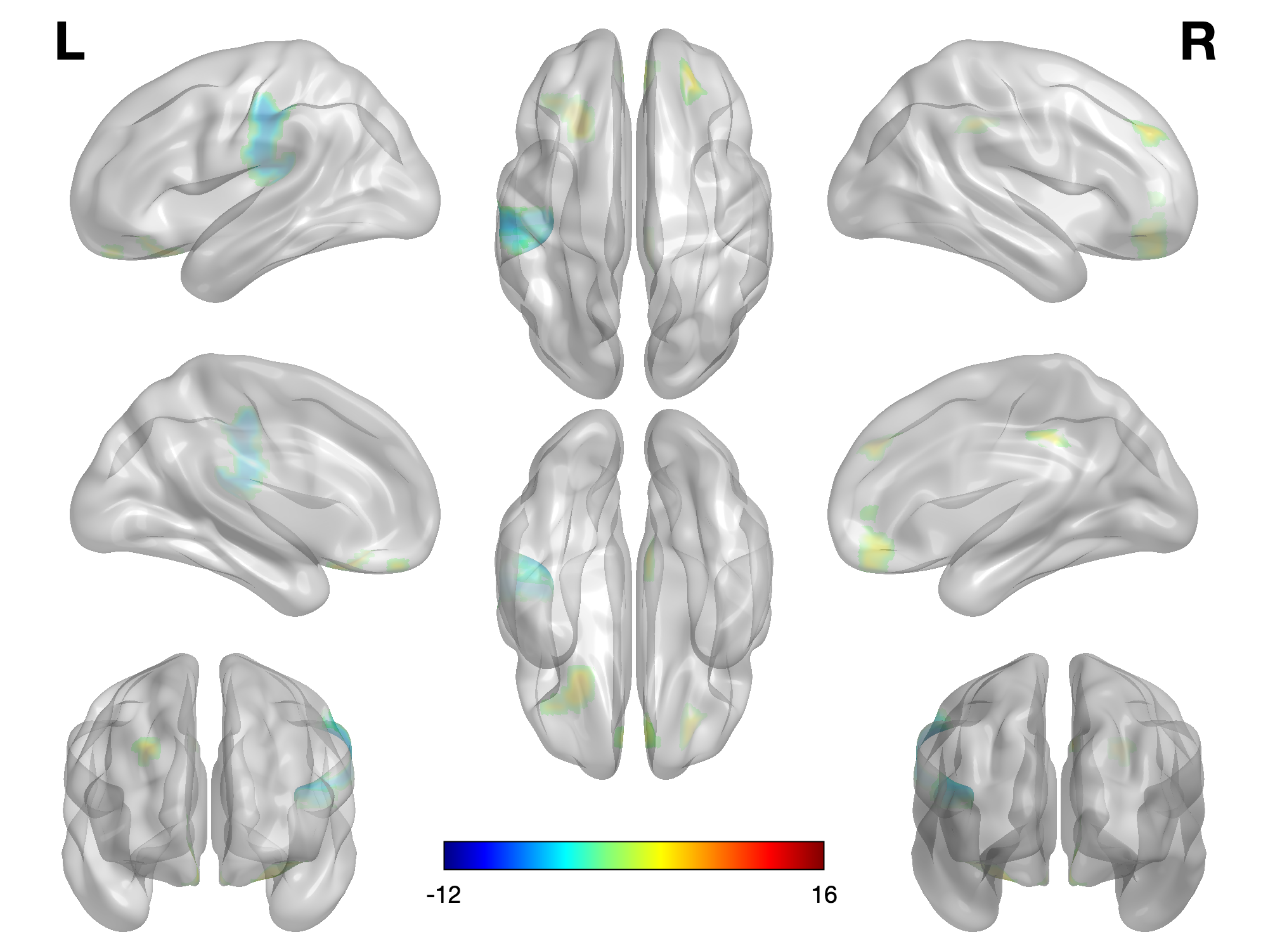


Table S2. Notable activation differences between successful neutral stop and failed neutral stop trials (*p* < 0.001 with cluster size > 50; *p*FWE values are after cluster-based FWE 0.05 correction).

| Hemisphere | Region | Peak MNI coordinates | | | Voxels | *t* | *pFWE* |
| --- | --- | --- | --- | --- | --- | --- | --- |
|  |  | X | Y | Z |  |  |  |
| B | SMG | -48 | -30 | 33 | 603 | *-6.95* | *0.001* |

MNI, Montreal Neurological Institute; SMG, supramarginal gyrus; B, bilateral.

Figure S2. Notable brain areas showing activation differences between successful neutral stop and failed neutral stop trials (Cluster-based FWE 0.05 correction with cluster size > 50).


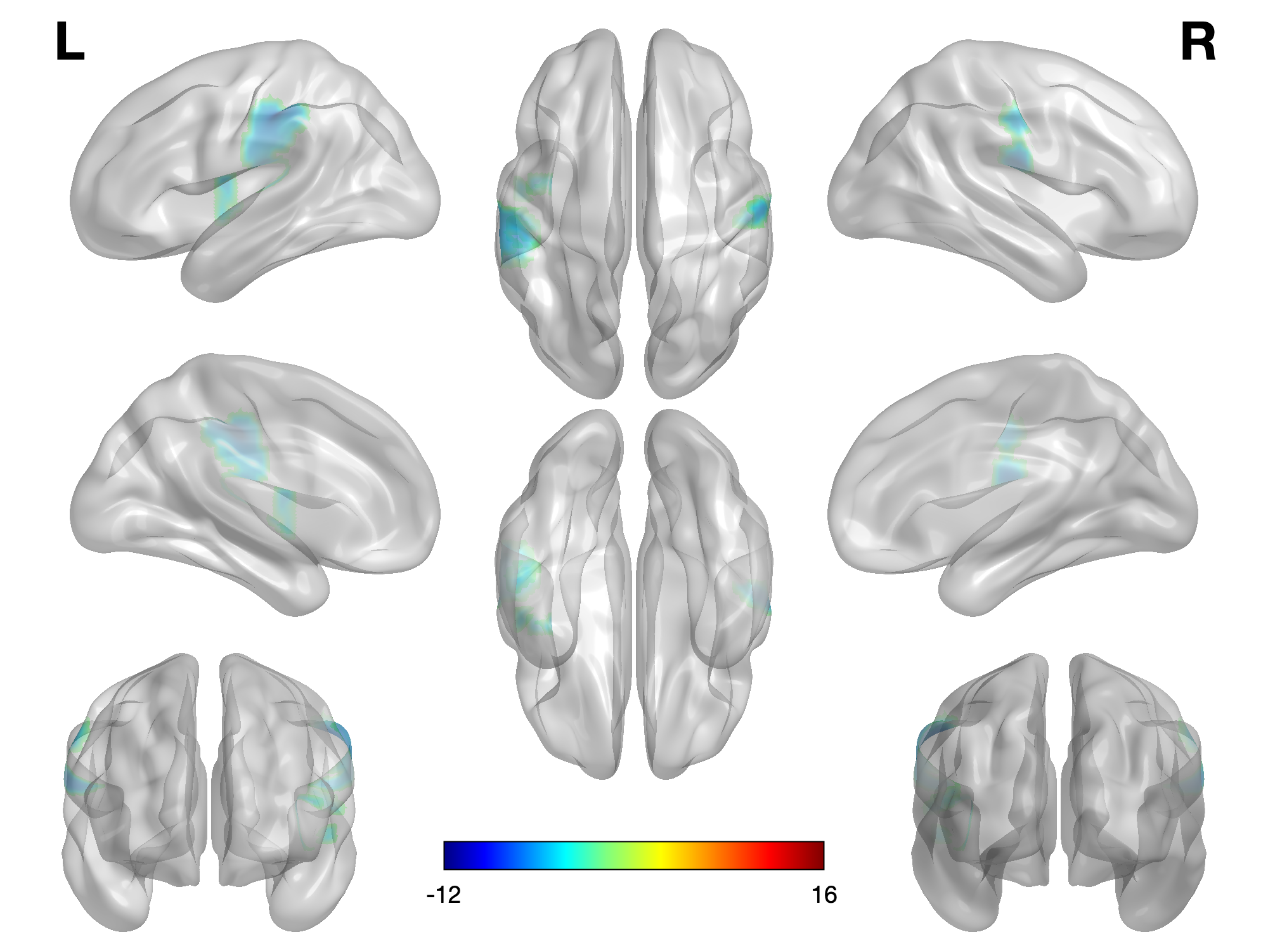


Table S3. Notable activation differences between successful sad go trials and baseline (*p* < 0.001 with cluster size > 50; *p*FWE values are after cluster-based FWE 0.05 correction).

| Hemisphere | Region | Peak MNI coordinates | | | Voxels | *t* | *pFWE* |
| --- | --- | --- | --- | --- | --- | --- | --- |
|  |  | X | Y | Z |  |  |  |
| B | MFG | -9 | -66 | 33 | 19068 | *11.19* | *0.001* |
| L | Putamen | -21 | 6 | -6 | 90 | *-6.25* | *0.01* |

MNI, Montreal Neurological Institute; MFG, middle frontal gyrus; B, bilateral; L, left.

Figure S3. Notable brain areas showing activation differences between successful sad go trials and baseline (Cluster-based FWE 0.05 correction with cluster size > 50).


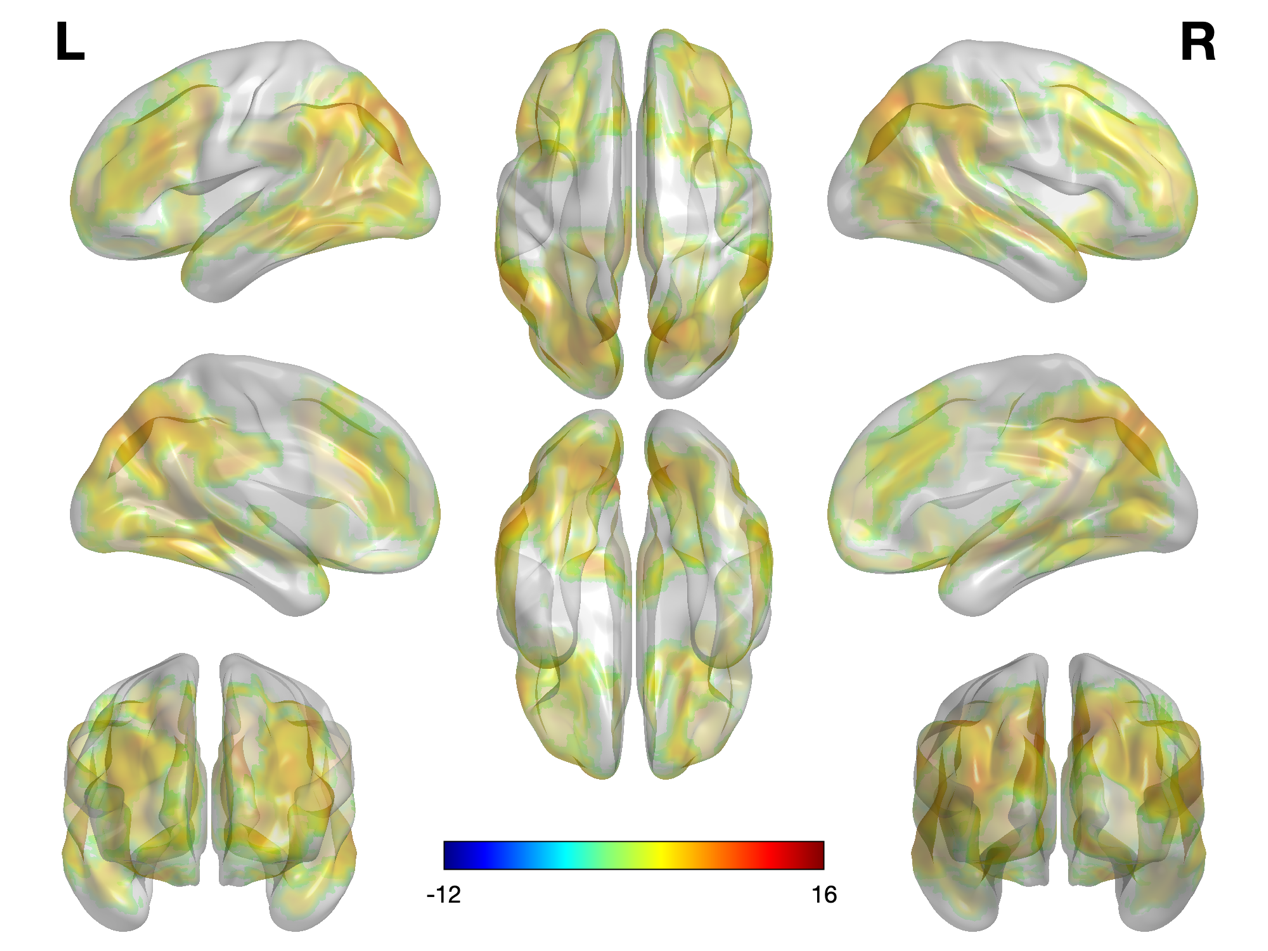


Table S4. Notable activation differences between successful sad stop trials and baseline (*p* < 0.001 with cluster size > 50; *p*FWE values are after cluster-based FWE 0.05 correction).

| Hemisphere | Region | Peak MNI coordinates | | | Voxels | *t* | *pFWE* |
| --- | --- | --- | --- | --- | --- | --- | --- |
|  |  | X | Y | Z |  |  |  |
| B | SOG | -15 | -75 | 39 | 15465 | *13.72* | *0.001* |
| R | IFG | 33 | 15 | 36 | 561 | *8.11* | *0.001* |
| L | Insula | -42 | -6 | 6 | 82 | *-6.49* | *0.03* |
| L | SMG | -51 | -24 | 42 | 1208 | *-11.88* | *0.001* |

MNI, Montreal Neurological Institute; SOG, superior occipital gyrus; IFG, inferior frontal gyrus; SMG, supramarginal gyrus; B, bilateral; L, left; R, right.

Figure S4. Notable brain areas showing activation differences between successful sad stop trials and baseline (Cluster-based FWE 0.05 correction with cluster size > 50).


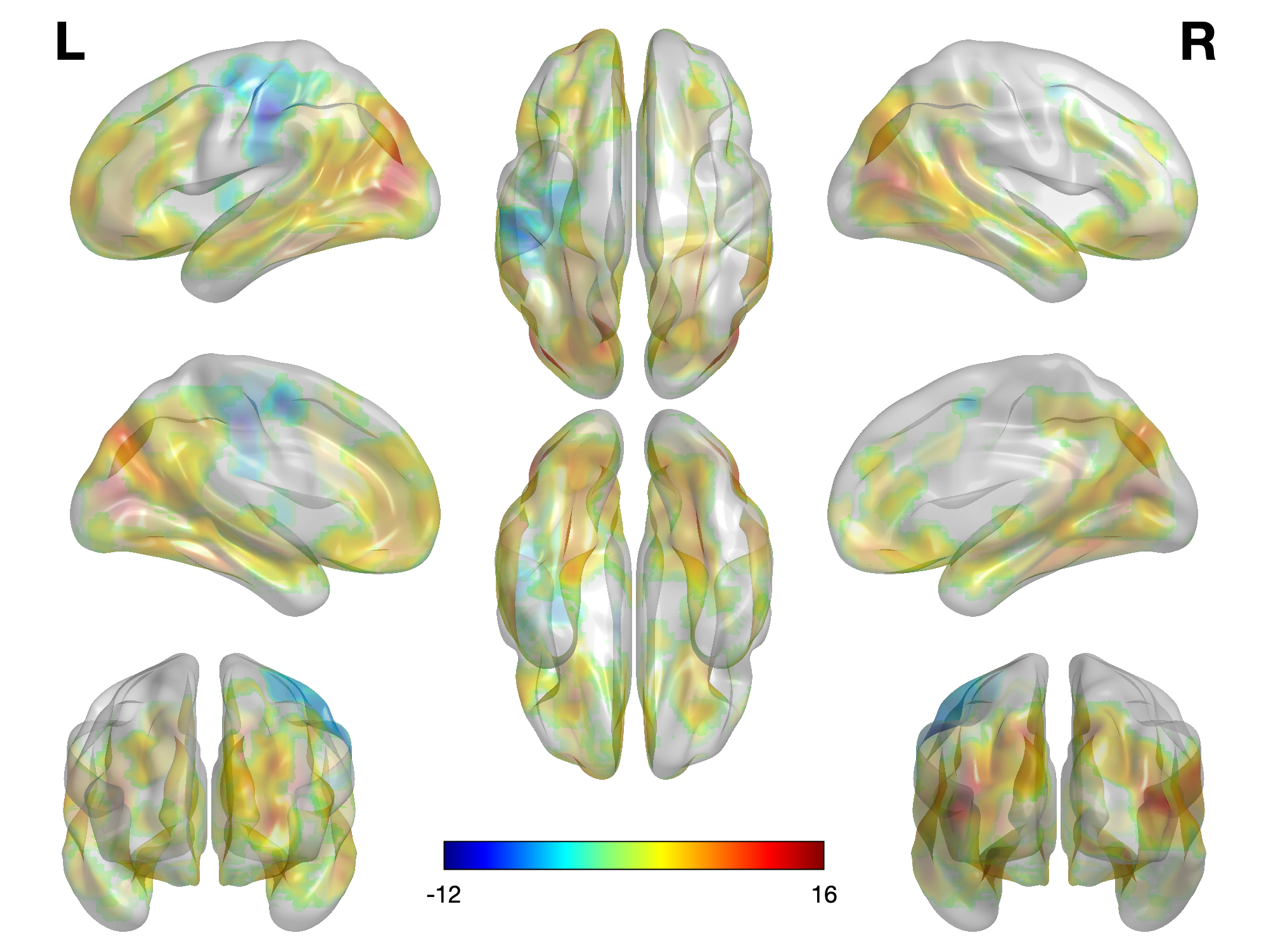


Table S5. Notable activation differences between successful neutral go trials and baseline (*p* < 0.001 with cluster size > 50; *p*FWE values are after cluster-based FWE 0.05 correction).

| Hemisphere | Region | Peak MNI coordinates | | | Voxels | *t* | *pFWE* |
| --- | --- | --- | --- | --- | --- | --- | --- |
|  |  | X | Y | Z |  |  |  |
| B | MFG | 15 | -69 | 39 | 21104 | *15.84* | *0.001* |

MNI, Montreal Neurological Institute; MFG, middle frontal gyrus; B, bilateral.

Figure S5. Notable brain areas showing activation differences between successful neutral go trials and baseline (Cluster-based FWE 0.05 correction with cluster size > 50).


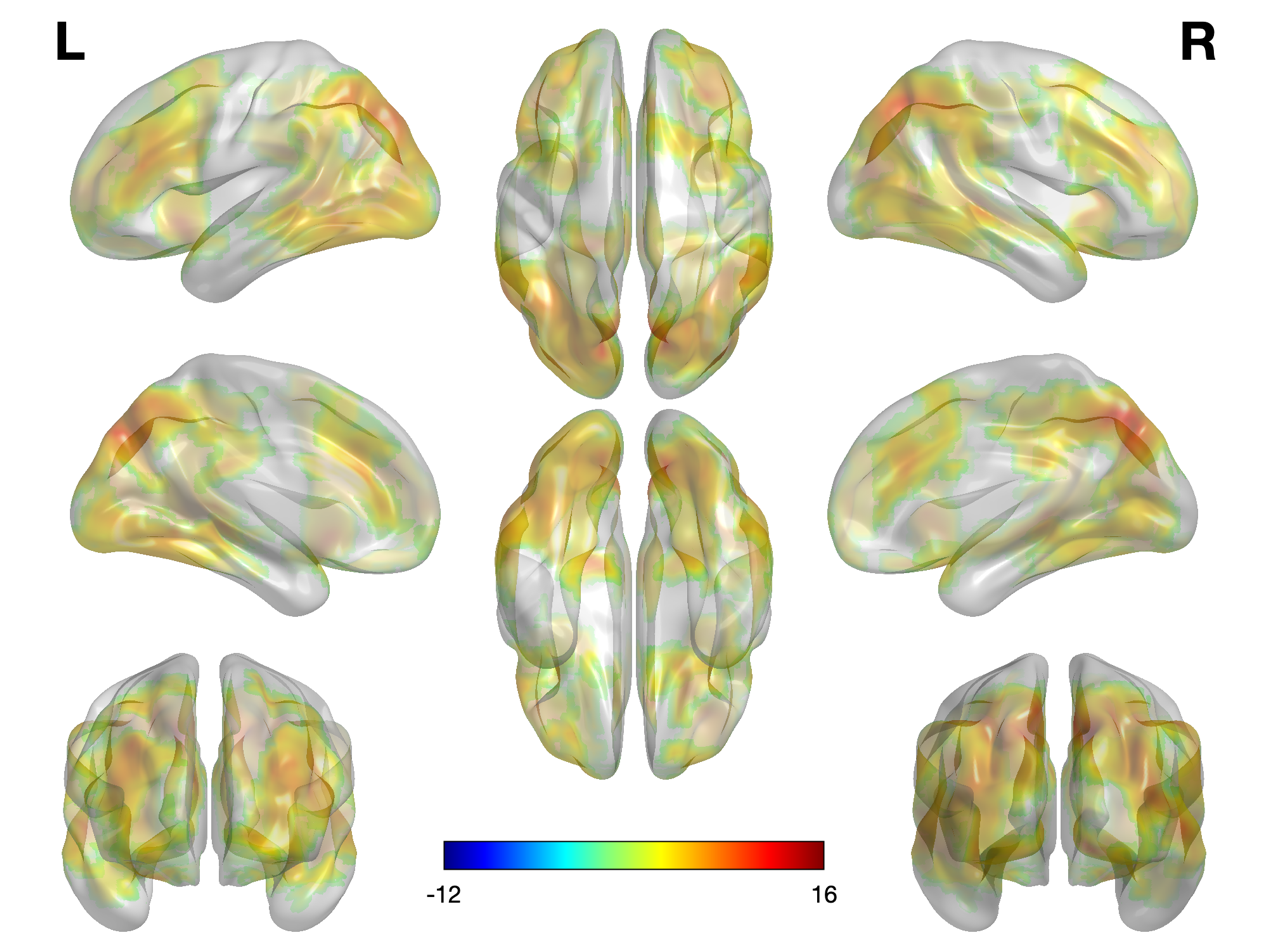


Table S6. Notable activation differences between successful neutral stop trials and baseline (*p* < 0.001 with cluster size > 50; *p*FWE values are after cluster-based FWE 0.05 correction).

| Hemisphere | Region | Peak MNI coordinates | | | Voxels | *t* | *pFWE* |
| --- | --- | --- | --- | --- | --- | --- | --- |
|  |  | X | Y | Z |  |  |  |
| B | IFG | -12 | -69 | 33 | 11487 | *14.01* | *0.001* |
| L | STG | -45 | -3 | 6 | 84 | *-5.56* | *0.04* |
| L | IPL | -48 | -27 | 42 | 1282 | *-8.97* | *0.001* |

MNI, Montreal Neurological Institute; IFG, inferior frontal gyrus; STG, superior temporal gyrus; IPL, inferior parietal lobule; B, bilateral; L, left.

Figure S6. Notable brain areas showing activation differences between successful neutral stop trials and baseline (Cluster-based FWE 0.05 correction with cluster size > 50).


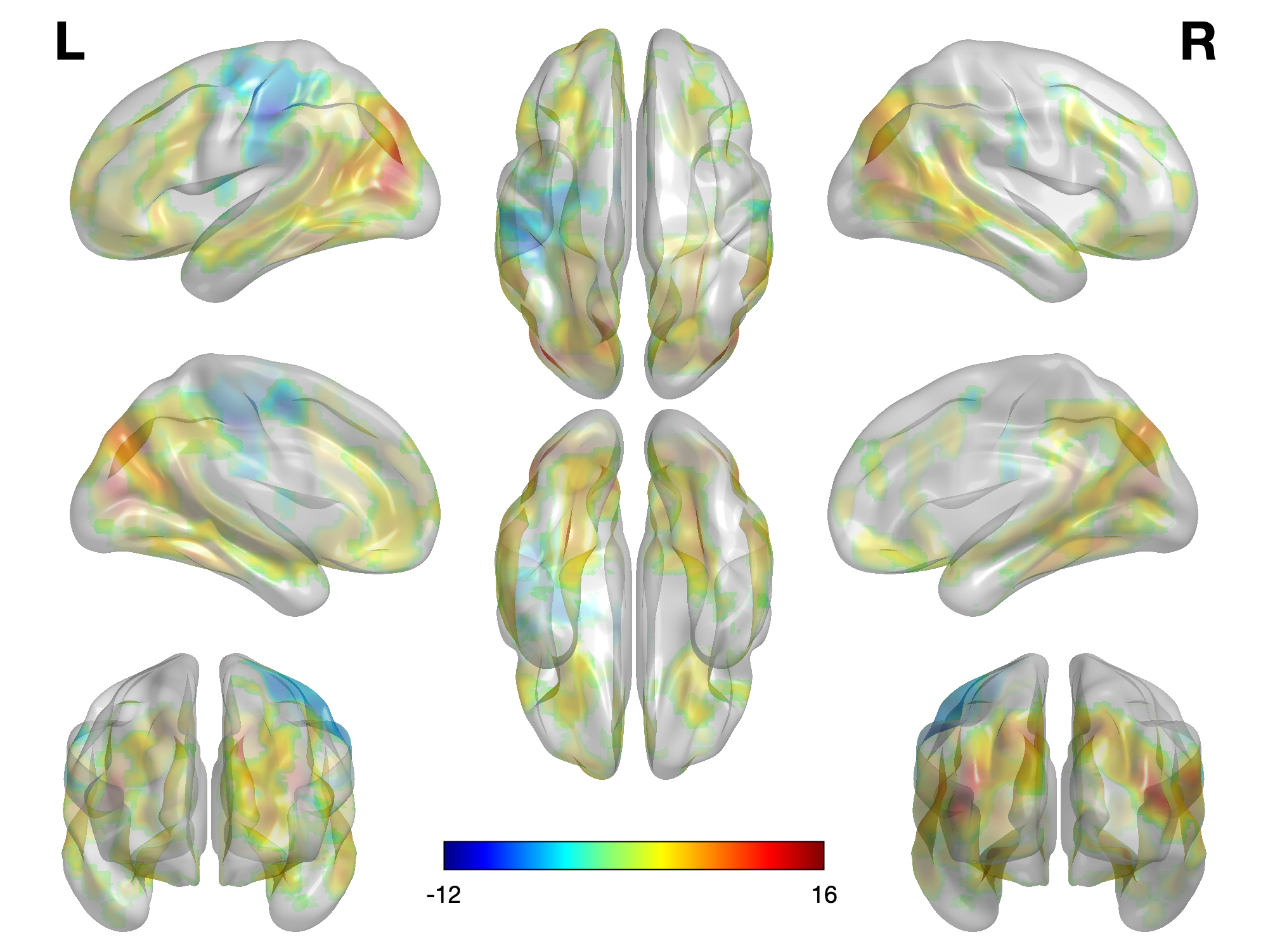


Table S7. Correlation between brain activity and neutral SSRT in successful neutral stop trials (*p* < 0.001 uncorrected with cluster size > 50; *p*FWE values are after cluster-based FWE 0.05 correction; the result cannot pass the FWE 0.05 correction).

| Hemisphere | Region | Peak MNI coordinates | | | Voxels | *t* | *pFWE* |
| --- | --- | --- | --- | --- | --- | --- | --- |
|  |  | X | Y | Z |  |  |  |
| R | Precuneus | 12 | -63 | 51 | 53 | *5.46* | *0.13* |

MNI, Montreal Neurological Institute; R, right.

Figure S7. Notable brain areas showing correlation with neutral SSRT in successful neutral stop trials (p < 0.001 uncorrected with cluster size > 50).


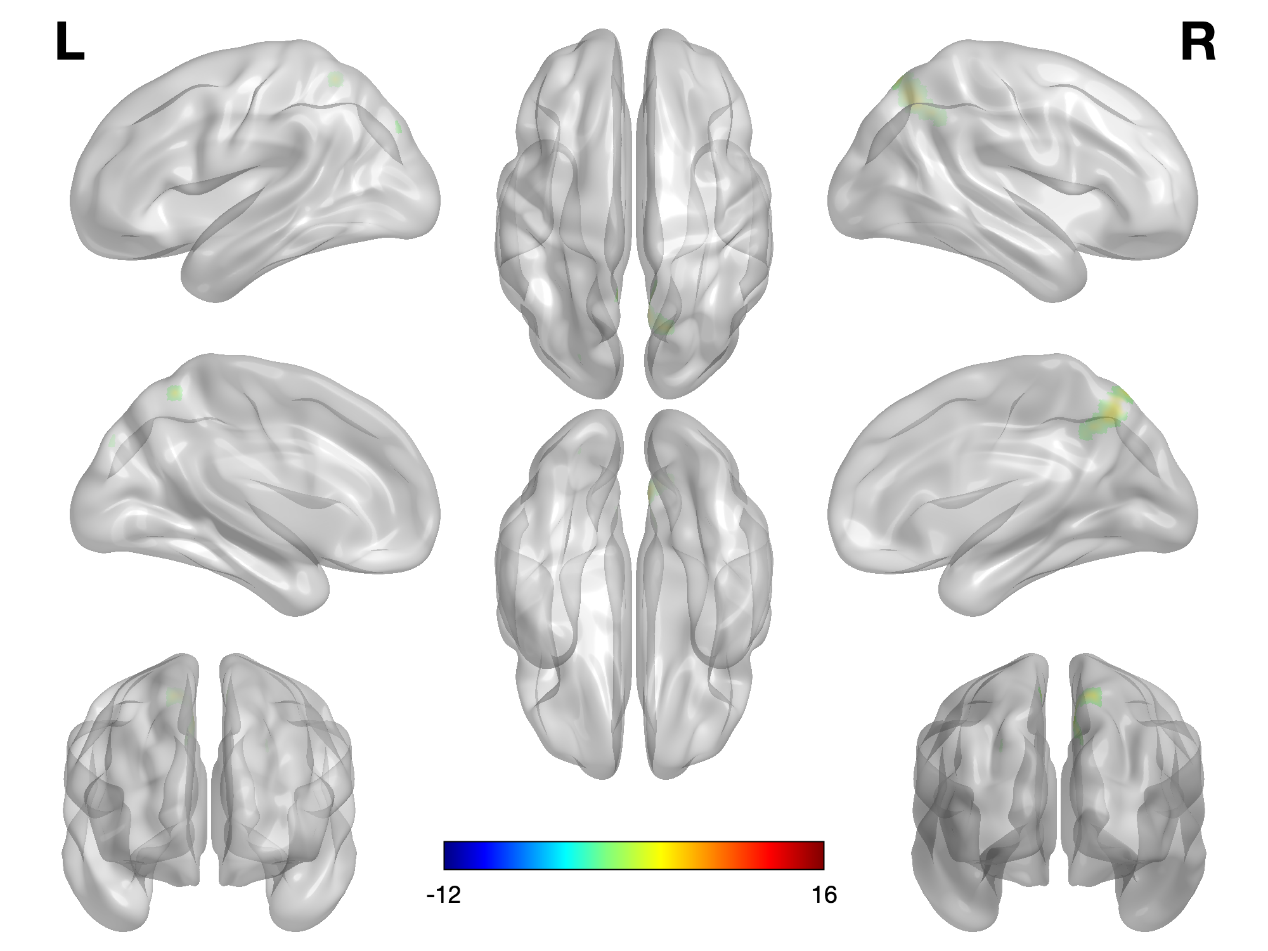


There is no notable correlation between brain activity and sad stop signal reaction time (SSRT) in successful sad stop trials.
